# Thyroid hormones and platelet activation in COVID-19 patients

**DOI:** 10.1007/s40618-022-01896-2

**Published:** 2022-09-05

**Authors:** E. Colonnello, A. Criniti, E. Lorusso, M. Curreli, M. Santulli, A. Angeloni, L. Gnessi, O. Gandini, C. Lubrano

**Affiliations:** 1 Department of Experimental Medicine, Sapienza University of Rome, Rome, Italy; 2 Department of Molecular Medicine, Sapienza University of Rome, Rome, Italy

**Keywords:** Thyroid, Platelets, COVID-19, Euthyroid sick syndrome, Non-thyroidal illness

## Abstract

**Purpose:**

To retrospectively describe the association between thyroid hormones (TH) and platelet activation, as represented by mean platelet volume (MPV), in a cohort of patients hospitalized for COVID-19 with no known thyroid disease, and to correlate these data with the severity of COVID-19 and the occurrence of death/ARDS (Acute Respiratory Distress Syndrome).

**Methods:**

103 patients with real-time polymerase chain reaction (RT-PCR) testing-confirmed COVID-19 and hospitalized were enrolled. Serum samples were collected from patients upon admission before starting any treatment. Chi-squared test was used to determine the association between euthyroid sick syndrome (ESS) and COVID-19 severity. Multivariate logistic regression was performed to evaluate the best independent predictors of COVID-19 deaths/ARDS.

**Results:**

39/103 (37.9%) of patients were found to have ESS, and this condition was an independent predictor for the severity of COVID-19 (*p* = 0.003). Lower TSH and lower FT3/FT4 ratio correlated with higher MPV (*p* = 0,001 and *p* = 0.010), with an opposite trend with respect to what has been documented in non-COVID patients. Increasing MPV and lower FT3 significantly increased the risk, in COVID-19 patients, of an adverse outcome of death/ARDS.

**Conclusion:**

Increased platelet activation, as represented by increased MPV, has already been reported to correlate with COVID-19 severity, possibly as a consequence of cytokine release. We demonstrated, in a cohort of 103 patients with COVID-19, that MPV is inversely correlated to TH levels, in particular in the case of ESS, where downregulation of TH axis may occur in case of systemic cytokine inflammation and more severe outcomes (death/ARDS). That ESS itself may directly cause platelet activation, as demonstrated by higher MPV in these patients, is an interesting hypothesis which deserves further investigation.

## Introduction

**Fig. 1:**
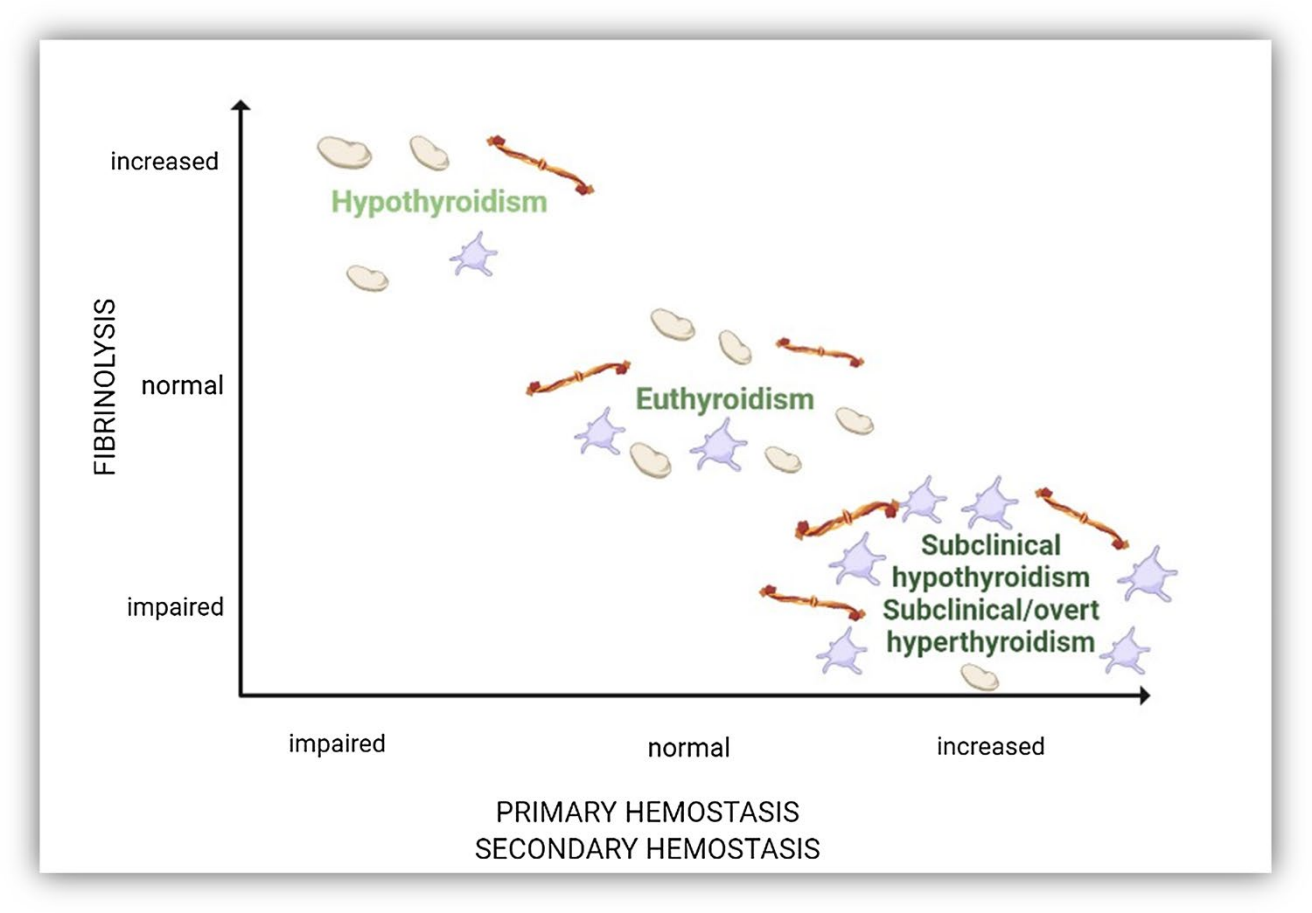
Thyroid dysfunction and hemostasis (readapted from [1])

Thyroid dysfunction has been associated with a variety of abnormalities in coagulation. Although limited, the published studies suggest that patients with either hyperthyroidism and subclinical hypothyroidism have an increased thrombotic risk, whereas patients with overt hypothyroidism have a bleeding tendency, explained mainly by abnormalities of primary hemostasis resembling acquired von Willebrand disease [10] (Fig. 1). Thyroid hormones (TH) indeed have an effect on coagulation both via genomic pathways mediated by a nuclear TH receptor[11], and also via non-genomic mechanisms initiated at the receptor for L-thyroxine (T4) on platelet integrin αvβ3, or vitronectin receptor, with prothrombotic action [11, 12]. Increased levels of procoagulant factors, such as F VIII, F IX, fibrinogen, and Von Willebrand factor (vWF), have been found in hyperthyroid patients, with high levels of free Thyroxine (FT4) being associated with the risk of venous thrombosis, up to an odds ratio of 2.2 [13]. Also, increased platelet function has been demonstrated in patients with overt hyperthyroidism, compared to euthyroid ones, with time of platelet plug formation being shortened by therapy with thyroid-inhibitor thiamazole [14]. On the other hand, also subclinical hypothyroidism (SCH), defined as an increased thyroid-stimulating hormone (TSH) level with a normal FT4 level, has been associated with hypercoagulability [15–17]. In particular, in several cross-sectional studies, mean platelet volume (MPV) has a significant higher value in patients with SCH compared to euthyroid ones [18–20], although this has not been confirmed by larger-scale studies [21]. MPV indicates the mean platelet size and reflects the platelet production rate and activation [22]. Higher MPV has been correlated to a variety of pathological diseases [23], as a marker of severity, including the disease caused by severe acute respiratory syndrome coronavirus 2 (SARS-CoV-2) infection. Severe coronavirus disease 2019 (COVID-19 disease) has been associated with the increased production of larger, younger platelets [24], with higher MPV being correlated to a more severe course of the disease [25] and an increased risk of mortality [26]. In these patients, MPV may reflect the hyper-inflammatory state caused by the cytokine storm secondary to the infection, and responsible for many COVID-19-associated deaths [27]. Indeed, SARS-CoV-2 exhibits a marked respiratory tropism causing mainly interstitial pneumonia and acute respiratory distress syndrome (ARDS), but is also able to induce a severe systemic inflammation, leading to multi-organ dysfunction and even death in subjects with high-risk factors (i.e., old age, male gender, obesity, diabetes, and cardiovascular comorbidities) [1–3]. More severe manifestations of COVID-19 are associated with an excessive immune and inflammatory response, characterized by elevated serum levels of C-Reactive Protein (CRP), D-Dimer (D-D), Lactate Dehydrogenase (LDH), Neutrophil-to-Lymphocyte ratio (NLR), ferritin and several pro-inflammatory cytokines, the so-called “cytokine storm”[4, 5]. Apart from the typical lung involvement, patients with COVID-19 can experience several extra-pulmonary manifestations, including cardiovascular and neuromuscular disorders, renal failure, coagulopathies (thrombosis, disseminated intravascular coagulation, anemia, and thrombocytopenia), and also endocrinopathies. Preexisting endocrinopathies may worsen COVID-19 prognosis [6–8]. Thyroid gland involvement has been emerging as a quite common event in the course of COVID-19 [9], via both a direct and an indirect mechanism of SARS-CoV-2 infection that can render the thyroid dysfunctional [28]. The first one is mainly mediated by the direct expression on the thyroid gland surface of factors facilitating viral entry (ACE2), while the second, indirect one, is mainly represented by the potent cytokine storm elicited by the infection.

Hence, given the well-known correlation between COVID-19 and, respectively, thyroid gland involvement on one side and increased platelet function on the other side, since the relationship between the latter two is still discussed, by means of a retrospective study on patients hospitalized for COVID-19 without known thyroid diseases, we hypothesized a possible association between TH levels and MPV values parallel to COVID-19 severity.

## Methods

This retrospective study included 103 patients with real-time polymerase chain reaction (RT-PCR) testing-confirmed COVID-19 admitted to the Policlinico Umberto I hospital from April 2020 to March 2021. Demographic, epidemiological, clinical, laboratory, radiological, treatment, and outcome data were collected, and patients without a history of thyroid disease who had a thyroid function test at admission were enrolled. Patients underwent a routine laboratory screening at the entry of the Emergency Department including C-reactive protein (CRP), ferritin. Serum samples were collected from patients upon admission before starting any treatment. Patients with uncontrolled diabetes mellitus or hypertension or in treatment with diuretics, beta blockers, corticosteroids, anti-lipidemic agents which have effects on both platelets and thyroid function tests were excluded.

All patients underwent high-resolution chest computed tomography (CT) to evaluate the presence of interstitial pneumonia and its severity. Patients were classified as (1) no pneumonia if there was no radiological sign of pneumonia, (2) mild pneumoniae if there was only interstitial involvement without consolidation, (3) moderate pneumoniae if there was interstitial involvement with consolidation in less of 50% of lung parenchyma and (4) severe pneumoniae if there was interstitial involvement and consolidation in more than 50% of lung parenchyma.

Based on the severity of pulmonary impairment in Computer Tomography (CT) scan and respiratory failure in need of mechanical ventilation, patients were further divided into 4 groups according to the WHO guidelines updated in November 2021 [29]: patients with no CT scan alterations or hypoxia (Group 0-mild); patients with CT scan signs, suggestive of pneumonia with no oxygen supplementation (Group 1-moderate); patients with CT scan signs, suggestive of pneumonia plus oxygen supplementation (Group 2-severe) and patients with CT scan changes, suggestive of ARDS plus intensive care unit (ICU) admission (Group 3-critical). After the initial evaluation and management, patients were discharged in home isolation or were hospitalized in low, medium or sub-intensive/intensive care units according to medical needs. All patients were followed up to 60 days after the Emergency Department admission. Patients were further grouped following the occurrence within 2 months from admission of death/ARDS (Acute Respiratory Distress Syndrome) or not. Non-thyroidal illness syndrome (NTIS) and euthyroid sick syndrome (ESS) were defined as serum FT3 < 2.3 pg/ml with low or normal levels of TSH. Statistical analysis was performed using StatSoft, Inc. (2014) (data analysis software system), version 12. www.statsoft.com. Continuous variables are reported as mean and standard deviation or median and interquartile range depending on variable distribution. Squared linear regressions between continuous variables were always corrected for sex and age. Chi-squared test was used to determine the association between ESS and COVID-19 severity. Multivariate logistic regression was performed to evaluate the best independent predictors of COVID-19 deaths/ARDS.

## Results

103 patients were included in the study, 57 females and 46 males. The mean age of the patients was 59.18 ± 17.18 years. The mean MPV was 9.04 ± 1.27 fL. The mean levels of TSH, free triiodothyronine (FT3) and FT4 were, respectively 1.69 ± 1.14 µU/ml (normal range 0.27–4.2); 2.50 ± 0.63 pg/ml (normal range 2–4.4); 1.43 ± 0.54 ng/dl (normal range 0.93–1.97).

14 patients showed no CT scan alterations or hypoxia (Group 0-mild); 7 patients were with CT scan signs suggestive of pneumonia with no oxygen supplementation (Group 1-moderate); 33 patients with CT scan signs suggestive of pneumonia plus oxygen supplementation (Group 2-severe) and 49 patients with CT scan changes suggestive of ARDS plus intensive care unit (ICU) admission (Group 3-critical). Patients were further grouped following the occurrence within 2 months from admission of death/ARDS—33 patients—or not—70 patients.

**Fig. 2:**
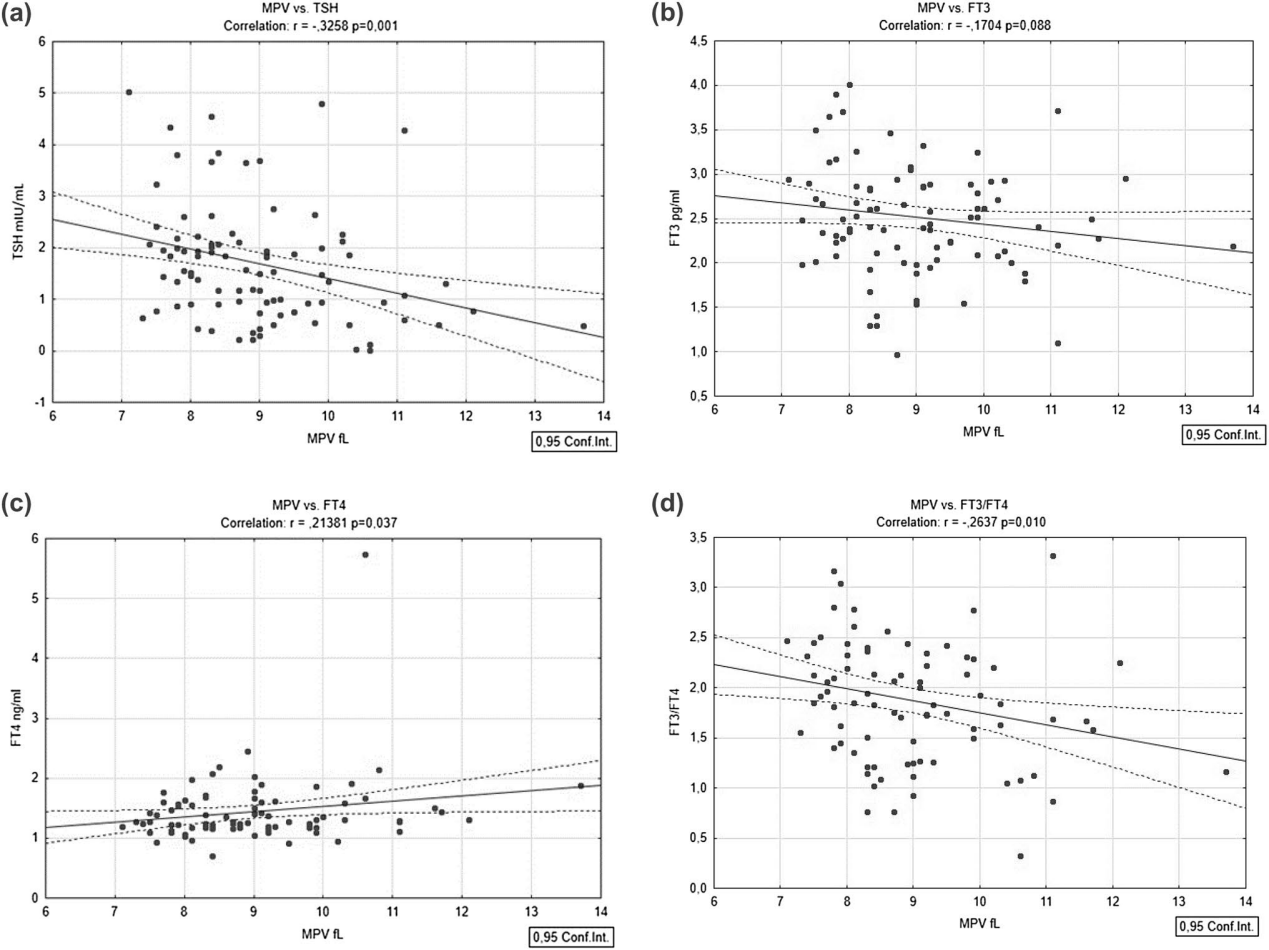
Scatter plot of **a** TSH **b** FT3 **c** FT4 and **d** FT3/FT4 values of the 103 patients with respect to MPV

Linear regression analysis was performed to evaluate the relationships among TH levels and MPV, with results shown in Fig. 2. All the regressions were adjusted for age and sex. FT3 showed a trend of negative correlation with MPV (*r* =  − 1704 *p* = 0.088), not statistically significant. FT4 was positively correlated with MPV (*r* = 2138, *p* = 0.037). Both TSH and FT3/FT4 ratio had a statistically significant inverse correlation with MPV (respectively, *r* =  − 3258; *p* = 001 for TSH; *r* =  − 2637, *p* = 010 for FT3/FT4).

**Fig. 3:**
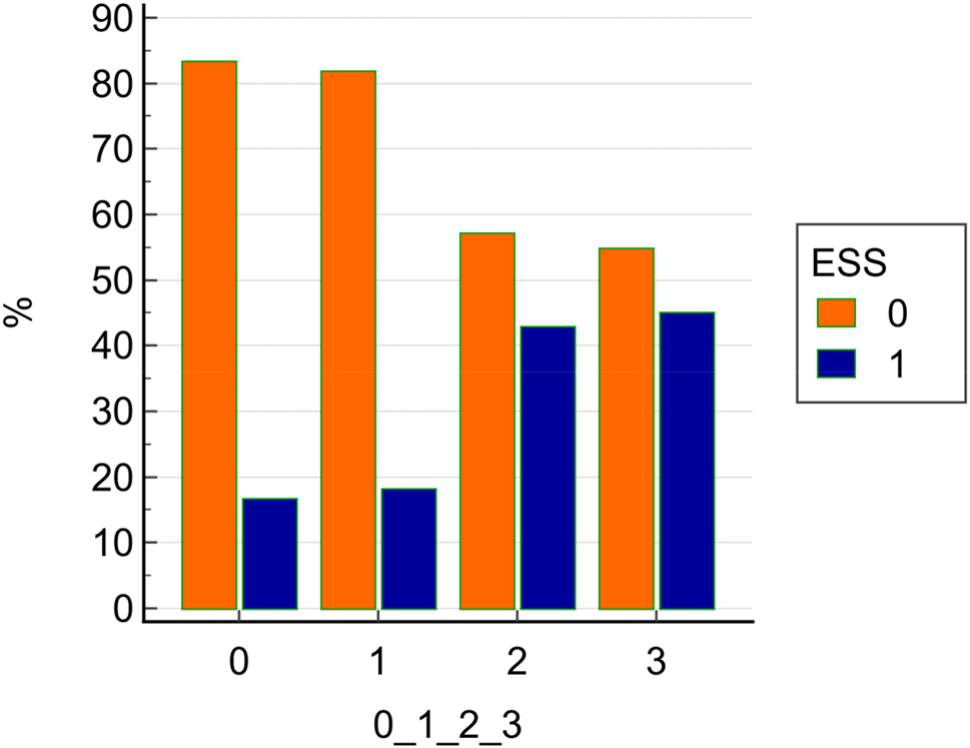
Chi-squared test. Percentages of patients with (blue) or without (orange) euthyroid sick syndrome (ESS), in the different categories of severity. 0 mild course: no CT signs, 1 moderate course: CT signs + no O2 supplementation, 2 severe: CT signs + O2 supplementation, 3 critical: ARDS CT signs + Intensive Care Unit

ESS was found in 39 patients (37.9%) 23 females, 16 males. Hence, based on this finding, patients were further divided into two groups according to serum FT3 values: the ESS group and the non-ESS group. Student’s T comparison revealed that ESS patients were, on average, older with respect to non-ESS patients (*p* < 0.001), having a higher MPV value (*p* = 0.027), a lower TSH, FT3 and FT3/FT4 ratio (*p* < 0.001) (Table 1). χ^2^ analysis revealed that ESS was more frequent with increasing severity of COVID-19 (*p* = 0.03) (Fig. 3).

**Table 1:** Student’s *T* test for comparison between ESS patients (39) and no-ESS patients (64)

Variable	Mean no ESS	Std.Dev no ESS	Mean no ESS	Std.Dev ESS	t value	df	*p*
Age (years)	53.18	16.63	66.90	14.55	− 4.17	94	0.000
PLT(× 10^3^/mm^3^)	220.03	95.04	202.05	100.10	0.88	93	0.378
MPV (fL)	8.74	1.20	9.36	1.49	− 2.25	93	0.027
FT3 (pg/dL)	2.89	0.45	1.89	0.35	11.74	94	0.000
FT4 (ng/dL)	1.35	0.28	1.51	0.77	− 1.45	92	0.150
TSH (mIU/mL)	2.04	1.21	1.22	0.86	3.62	94	0.000
FT3/FT4	2.21	0.49	1.38	0.42	8.44	92	0.000
Number of comorbidities	0.65	0.72	1.36	1.51	− 3.18	100	0.002

One-way analysis of variance (Table 2) revealed that FT3 decreased with increasing severity of COVID-19 (*p* = 0.03) while both age and MPV increased with increasing severity (*p* = 0.03).

**Table 2:** Analysis of variance

Variable	0	1	2	3	*p*
Age (years)	52.28 ± 20.14	55.12 ± 18.89	61.05 ± 15.56	64.07 ± 15.77	0.030
PLT (× 103/mm3)	205.28 ± 48.90	213.65 ± 72.91	22.52 ± 80.01	211.11 ± 108.16	0.895
MPV (fL)	8.34 ± 0.78	8.83 ± 0.92	9.00 ± 1.10	9.31 ± 1.46	0.032
FT3 (pg/dL)	2.94 ± 0.58	2.57 ± 0.19	2.52 ± 0.63	2.36 ± 0.64	0.026
FT4 (ng/dL)	1.30 ± 0.18	1.09 ± 0.11	1.54 ± 0.84	1.44 ± 0.33	0.218
TSH (mIU/mL)	1.83 ± 1.14	2.39 ± 1.40	1.72 ± 1.15	1.53 ± 1.10	0.289
FT3/FT4	2.26 ± 0.54	2.38 ± 0.18	1.86 ± 0.68	1.69 ± 0.55	0.003
Number of comorbidities	0.44 ± 0.51	0.33 ± 0.59	0.78 ± 0.84	1.43 ± 144	0.000

0 mild course: no CT signs, 1 moderate course: CT signs + no O2 supplementation, 2 severe: CT signs + O2 supplementation, 3 critical: ARDS CT signs + Intensive Care Unit

*T* test for independent samples was performed to evaluate differences between ARDS/Death patients and the control group (Table 3).

**Table 3:** *T* test for independent samples by COVID-19 severity (ARDS/Death vs. recovery)

Variable	Death/ARDS 1 = yes 0 = no						
	Mean 0	Std.Dev 0	Mean 0	Std.Dev.1	t value	df	*p*
SEX 1 = F 2 = M	1.47	0.50	1.59	0.50	− 1.35	136	0.179
AGE years	57.38	16.91	68.31	12.86	− 3.94	136	0.000
PLT (× 103/mm3)	214.46	83.28	215.18	105.21	− 0.04	132	0.965
MPV (fL)	8.77	1.03	9.62	1.50	− 3.83	127	0.000
FT3 (pg/ml)	2.64	0.60	2.18	0.58	3.54	100	0.001
FT4 (ng/ml)	1.43	0.61	1.46	0.37	− 0.28	94	0.783
TSH (mIU/mL)	1.85	1.20	1.35	0.94	2.10	101	0.039
FT3/FT4	2.00	0.60	1.58	0.57	3.20	93	0.002
Number of comorbidities	0.84	1.01	1.81	1.75	− 3.56	141	0.000

Multivariate logistic regression to evaluate independent predictors of COVID-19 severity (deaths/ARDS) showed that MPV (fL) was significantly positively related to the outcome, while FT3 (pg/ml) was negatively related (Table 4). In particular, when adjusted for comorbidities, increasing MPV and lower FT3 significantly increase the risk, in COVID-19 patients, of an adverse outcome of death/ARDS (*p* = 0.02 and *p* = 0.05, respectively).

**Table 4:** Multivariate logistic regression to evaluate independent predictors of COVID-19 outcome ((ARDS/Death vs. recovery)

Variable	Coefficient	Std. Error	Wald	*p*	Odds ratio	95% CI
Age years	0.01679	0.01933	0.75505	0.384	1.0169	0.97864–1.05673
Sex	1.05455	0.56986	3.44032	0.063	2.87068	0.92852–8.87525
FT3 ng/dL	− 0.95485	0.48075	3.94474	0.047	0.38486	0.14818–0.99957
MPV fL	0.55081	0.23311	5.58329	0.018	1.73466	1.09202–2.75550
Number of comorbidities	0.59448	0.32748	3.29530	0.069	1.81210	0.94586–3.47167
Constant	− 6.82757	2.93610	5.40743	0.020	0.00108	0.00000–0.36836

## Discussion

TH exerts effects on different levels of the hemostatic system (Fig. 1). It has been demonstrated that physiological concentrations of T4, but not T3, activate human platelets resulting in ATP release and aggregation [30]. Hyperthyroidism is associated with an increase in vWF activity levels [31] which may consecutively lead to increased cardiovascular risk [32]. On the other hand, also SCH has been found to be associated with hypercoagulability, reversible by 6 months of L-T4 treatment [17]. In particular, in several cross-sectional studies, MPV, a marker of platelet activation, has a statistically significant higher value in patients with SCH compared to euthyroid ones [18–20], suggesting a possible prothrombotic effect of elevated thyrotropin (TSH) [18]. Indeed, the association between SCH and coronary artery disease [33–35], as well as endothelial dysfunction [36, 37], is established, with an increased risk of cardiovascular events and mortality in those with higher TSH levels, particularly in those with a TSH concentration of 10 mIU/L or greater.

Platelets have a disk-shaped appearance in a quiescent state, but as they activate, they undergo a disk-to-sphere transformation with the development of pseudopodia, causing a subsequent increase in size (Fig. 1), which is mirrored by an increase in MPV [38]. Larger platelets are more metabolically and enzymatically active than smaller platelets, and have been found in several diseases, such as coronary arterial disease [39], insulin resistance [40], diabetes [41], dyslipidemia [42] and several inflammatory diseases [23]. COVID-19 represents the prototype of severe inflammatory disease, with the potent cytokine storm elicited by the infection being one of the main responsible of the COVID-19-associated deaths, along with pulmonary function impairment. SARS-CoV-2 is in fact a pleiotropic virus, known to exert its effects in many body organs other than the respiratory tract, such as the brain, heart, kidney [43], penis [44], and, not ultimately, thyroid [45]. In particular, whereas high TSH levels were reported only in up to 8% of patients with COVID-19, the most frequently reported abnormality, in 15 up to 56% of patients with COVID-19, is ESS [45–47] with patients typically presenting low or low-normal plasma T4, low plasma T3, increased plasma reverse T3 (rT3) concentrations in the absence of a rise in TSH. ESS has been found to occur in many critical illnesses [48], featuring different phases. In the acute one, usually changes in TH-binding predominantly occur, along with peripheral TH uptake, and alterations in the expression and activity of the type-1 and type-3 deiodinases. In particular, the former one, being the dominant enzyme driving the conversion of T4 to T3, is suppressed, while the second one, which contributes to the rise in rT3, is increased. Differently, in the prolonged phase of illness, there are alterations in the central regulation of the thyroid axis, with hypothalamic thyrotropin-releasing hormone (TRH) expression being suppressed, which explains reduced TSH secretion and therefore reduced TH release. ESS has been frequently reported in critically ill patients, where it represents more like an adaptive mechanism than a true dysfunction, in the sense that lowering the availability of the active T3 reduces energy expenditure to limit catabolism [47, 49]. As a matter of fact, the same mechanism appears to occur in healthy, fasting subjects [50].

Studies on COVID-19 patients found that FT3 and TSH were lower in the presence of more severe symptoms [28, 51, 52], while total T4 levels alone were not correlated with the gravity of the disease. This also occurred in our sample of patients, with ESS patients showing more severe signs of the disease with respect to non-ESS (Fig. 3) and more frequent occurrence of death/ARDS (Table 2, Fig. 5). Several studies have already correlated ESS to a poor outcome in patients with COVID-19 [49, 53, 54]. What it is interesting and relatively new in our study is that, in our sample of COVID-19 patients, other than with disease severity, lower TSH is correlated also with higher MPV. Our primary hypothesis is that, whether they already have an ESS, as demonstrated by low FT3 values found in 37.8% of patients, or they show a trend toward ESS, may partly explain such findings, as ESS usually occur in patients with acute, non-thyroidal, diseases, like sepsis, surgery or other types of physical stress, as a result of an adaptive mechanism to preserve energy. Hence, the higher the severity of COVID-19, the higher the degree of inflammation leading to hyperactive platelets as reflected by higher MPV, the higher the ESS, which in this case appears to be rather as a para-phenomenon of higher platelet activation. Indeed, the potent cytokine storm elicited by COVID-19 infection is able to over-activate the immune system with the subsequent massive production of inflammatory cytokines molecules [27, 55, 56]. This hyper-inflammatory state not only induces platelet activation [25, 55], but it is also responsible for severe damage to many vital organs [27]. The cytokines mainly involved in this process are IL1β, IL6, TNF-α, and IFN-γ, and, interestingly, some of them are also involved in immune-mediated thyroid diseases [56]. For instance, on the hypothalamus–pituitary–thyroid axis (HPT), they act by lowering TSH secretion, while, peripherally, they reduce the metabolism of TH via deiodinases by reducing the conversion of T4 to T3 and increasing the inactivation of both T4 and T3 [27], thereby possibly contributing to the ESS syndrome often found in COVID-19 patients. As a matter of fact, in patients with COVID-19 (and also in our sample), ESS occurs especially in those with higher clinical severity, who, therefore, may be in a state of higher systemic inflammation (as shown by higher MPV value) with respect to those with less severity. In particular, increasing MPV and lower FT3 significantly increase the risk of a poor outcome (*p* = 0.02 and *p* = 0.05, respectively). The hypothesis is that, in such patients, ESS may intervene precociously, acting as a sort of a “protective” condition to reduce catabolism in the setting of the hyper-inflammatory state driven by COVID-19, explaining the inverse relationship between TSH and MPV evidenced only in COVID-19 patients. On the other hand, a second, interesting hypothesis emerges from this study, that is, ESS itself may be the cause of abnormal platelet activation and higher MPV. Indeed, patients with nephrotic syndrome with ESS have been found to have abnormal platelet activation and increased platelet aggregation [57]. Pro-coagulative state associated with low T3 syndrome can be an important and previously undescribed mechanism that place patients with untreated ESS at high risk of thromboembolic complications. Indeed, it is well-known the association of ESS with greater disease severity, mortality, and unfavorable prognosis [58]. For instance, in patients with acute ischemic stroke, ESS was found to be an independent risk factor for hemorrhagic transformation [59]and poor functional outcome [60]. Moreover, ESS is independently associated with increased risks of all-cause mortality, cardiac mortality and major adverse cardiovascular events (MACE) in cardiovascular patients [61]. Whether supplementing thyroid hormones to raise FT3 levels into the normal range can help to ameliorate the outcomes of ESS patients is still unclear. However, considering a possible interaction of ESS with platelet activation and aggregation as a determinant of a worse prognosis in these patients, and to look at MPV as a simple and reliable marker of severity, can be an interesting field to explore. However, more clinical studies on patients with ESS, also due to other clinical conditions, and platelet activation are needed to establish this relationship.

## Conclusion

This retrospective study aimed to assess the relationship between TH and platelet activation, as described by MPV, in a sample of hospitalized patients for COVID-19. 39 patients (37.9%) were found to have ESS, and, interestingly, their condition was a predictor of the severity of COVID-19, as shown by a combination of clinical and serological parameters. In our sample, lower TSH and lower FT3/FT4 ratio also correlated with higher MPV, with an opposite trend with respect to what has been documented in non-COVID patients. Also, ESS correlated with an adverse outcome (death or ARDS). Whether, in such patients, ESS may be a worse prognosis factor causing platelet activation, other than a functional adaptation able to lower catabolism in the presence of severe cytokine inflammation (and platelet activation) is an interesting hypothesis that, however, needs to be tested in a larger-scale study.
